# Stenotrophomonas maltophilia Meningitis - A Case Series and Review of the Literature

**DOI:** 10.7759/cureus.11221

**Published:** 2020-10-28

**Authors:** Iffat Khanum, Aisha Ilyas, Farheen Ali

**Affiliations:** 1 Infectious Diseases, Aga Khan University Hospital, Karachi, PAK; 2 Medicine, Aga Khan University Hospital, Karachi, PAK

**Keywords:** nosocomial meningitis, stenotrophomonas meningitis, gram-negative meningitis

## Abstract

*Stenotrophomonas maltophilia* is an infrequent cause of acute bacterial meningitis and only a few cases have been reported in the literature. Infection is associated with morbidity and mortality, and its optimal management remains ill-defined. The aim of the current study is to review the management of *S. maltophilia* meningitis.

We described two cases of *S. maltophilia* meningitis following neurosurgical procedures. The first patient was a 60-year-old female. She was admitted to the hospital with a left basal ganglia bleed and underwent placement of an external ventricular drain for the treatment of hydrocephalus. She developed *S. maltophilia* meningitis 20 days after surgery. She was successfully treated with a combination of trimethoprim-sulfamethoxazole and intravenous colistin and the removal of the drain. She successfully underwent a ventriculoperitoneal (VP) shunt placement at the therapeutic midway point. The second patient was a 35-year-old male with a history of intracranial aneurysm bleeding. He had undergone a craniotomy and placement of a ventriculoperitoneal shunt two years previously. His shunt was replaced twice due to blockage. The last replacement had occurred 15 days prior to the development of meningitis. He was treated with a combination of trimethoprim-sulfamethoxazole and ceftazidime (as well as undergoing another shunt replacement) and experienced an excellent recovery.

*S. maltophilia* is a rare but important cause of nosocomial meningitis. It is strongly associated with prior hospitalization and neurosurgical intervention, which is also found in our case series. The management of *S. maltophilia* meningitis is a therapeutic challenge due to its high resistance to multiple antibiotics. Optimal therapy is based on antimicrobial sensitivity, and the trimethoprim-sulfamethoxazole-based combination has been shown to be successful. The duration of therapy is debatable, but like most gram-negative meningitis infections, therapy lasting up to three weeks appears to be adequate.

## Introduction

*Stenotrophomonas maltophilia*, initially known as *Bacterium bookeri*, was first isolated in 1943. It was periodically classified as a member of the genus *Pseudomonas*, then *Xanthomonas*, and finally *Stenotrophomonas* in 1993. The genus *Stenotrophomonas* currently consists of four species, only one of which is known to cause infection in humans. *S. maltophilia* is an aerobic, non-fermentative, gram-negative bacillus that can occur in almost any aquatic or humid environment, including drinking water supplies [1]. *S.maltophilia, *through its ability to form a biofilm, can easily adhere to the devices and surfaces in a hospital environment which not only helps in its transmission but also increases its resistance to many antibiotics.

Historically considered as an opportunistic pathogen, *S. maltophilia* is now recognized as an important nosocomial pathogen with a high mortality rate. Infections due to *S. maltophilia* are particularly common with immunosuppression, extended use of antibiotics, intensive care unit admission, prolonged hospital stay, and presence of indwelling devices. It is responsible for a range of infections like pneumonia, bacteremia, biliary sepsis, urinary tract infection, endocarditis, meningitis, skin and soft tissue infection, endophthalmitis, septicemia, catheter-related bloodstream infection, osteomyelitis, and joint infections [2].

Despite the fact that *S. maltophilia* can cause a variety of infections, it still remains an uncommon cause of meningitis [3]. Its management is challenging due to the resistance of *S. maltophilia* to multiple antimicrobial agents and different antimicrobial susceptibility among different strains. It is only reported in the form of brief case series or case reports in the literature [4].

We report two cases of *S. maltophilia* meningitis following neurosurgical interventions and discuss therapeutic advances based on a brief review of the literature.

## Case presentation

Case one

A 60-year-old female with uncontrolled hypertension, presented with basal ganglia bleed and obstructive hydrocephalus. She underwent emergency placement of an external ventricular drain (EVD). Twenty days following the procedure, she developed a fever with no overt systemic signs of infection. As part of the fever evaluation, a cerebrospinal fluid (CSF) sample was sent for analysis and culture. Her initial CSF analysis showed very low glucose levels and polymorphic pleocytosis with gram-negative bacilli seen on the CSF gram stain, thus confirming meningitis (Table 1). The gram-negative bacilli were later identified as *S. maltophilia *using the 5 analytical profile index (API) 20NE test. API 20NE is a biochemical panel for identification and differentiation of non-fastidious Gram-negative rods not belonging to the family Enterobacteriaceae. It is a well-established method for manual microorganism identification to the species level. Considering the high prevalence of multidrug-resistant gram-negative organisms like MDR *Acinetobacter* and* Klebsiella* spp. in our hospital, the patient was empirically started on intravenous meropenem (2 gm every eight hours) and polymyxin E (3 million units every eight hours) along with intrathecal polymyxin E (300,000 IU/day). Drug susceptibility on disc diffusion showed that the organism was only sensitive to trimethoprim-sulfamethoxazole (TMP-SXM) and colistin. Therefore, her treatment was modified on the third day of illness. Intravenous meropenem was stopped, colistin (both intravenous and intrathecal) was continued and TMP-SMX (15m/kg/d in three divided doses) was added. The patient underwent replacement of the EVD after four days of the modified therapy. Three days following the removal of the EVD and seven days after the start of the TMP-SMX-based combination regimen, her repeat CSF cultures were negative, with significant improvement in all the CSF indices (Table 1).

**Table 1:** Cerebrospinal fluid analysis Case 1 Normal values in cerebrospinal fluid (CSF); Glucose 40-70 mg/dl, Protein 15–40 mg/dl, WBC: white blood cell 0–5 , DLC  Differential leucocytes count

CSF Analysis	DAY 1	DAY 4	DAY 7
Glucose (mg/dl)	12	84	76
Protein (mg/dl)	88	78	47
WBC ( 0 – 5)	80	26	12
DLC %	90% neutrophils	10% neutrophils	5% neutrophils
Culture	S. maltophilia	No growth	No growth

The intrathecal antibiotics were stopped once the CSF culture (day seven) was negative, but systemic colistin was continued for two weeks and TMP-SMX for 21 days. Her initial blood cultures were reported as negative and all 11 of her subsequent CSF cultures remained negative. There was no neurological sequelae post-treatment and she successfully underwent a ventriculoperitoneal (VP) shunt placement at the therapeutic midway point.

Case two

A 35-year-old male underwent a craniotomy and VP shunt placement following an intracranial aneurysm bleed that had occurred two years previously. He required replacement of the VP shunt twice due to shunt blockage. The first replacement had been performed one year before and the second was performed two weeks prior to the onset of fever. He developed a fever with altered mental status i.e. drowsiness two weeks after the replacement of the last VP shunt. As part of the fever workup, his blood, urine, and CSF samples were sent for culture and analysis. His CSF gram stain showed gram-negative bacilli with very low CSF glucose levels, high protein levels and white cells, thus confirming a meningitis diagnosis (Table 2).

**Table 2:** Cerebrospinal fluid analysis Case 2 Normal values in cerebrospinal fluid (CSF); Glucose 40-70 mg/dl, Protein 15–40 mg/dl, WBC: white blood cell 0–5 DLC  Differential leucocytes count

CSF Analysis	DAY 1	DAY 4	DAY 15
Glucose (mg/dl)	16	23	78
Protein( mg/dl)	257	102	42
WBC ( 0 – 5)	131	75	10
DLC %	80% neutrophils	70% neutrophils	5% neutrophils
Culture	S. maltophilia	No growth	No growth

The bacilli were identified as *S. maltophilia* using the API 20NE test. The drug susceptibility on disc diffusion showed sensitivity to TMP-SMX and ceftazidime. He was initially started on intravenous meropenem (2 gm every eight hours) along with intravenous colistin, which was later modified to intravenous TMP-SMX 15mg/kg/d and ceftazidime (2 gm every eight hours) as per antibiotics sensitivities of *S. maltophilia* reported in CSF culture. Additional therapeutic measures included shunt removal. His initial blood cultures were reported negative after seven days. Clinical improvement was noted after 72 hours of treatment. Repeated CSF analyses showed gradual improvement, and all subsequent CSF cultures were negative (Table 2). VP shunt was replaced after two weeks of appropriate antibiotic therapy. He received therapy for 21 days with marked clinical recovery.

## Discussion

Based on different inclusion studies, the incidence of post neurosurgical meningitis has been reported to be between 0.3% - 8.9 %. Risk factors include neurosurgical procedures such as craniotomies, especially in the presence of EVD devices, prolonged hospital stays, prior antibiotic exposure, and the existence of chronic illnesses such as diabetes and malignancies. Among other gram-negative pathogens such as* Escherichia coli, Acinetobacter *spp.,* Pseudomonas aeruginosa*, and *Klebsiella pneumoniae*, *S. maltophilia* remains a rare cause of healthcare-associated meningitis and only a few cases have been reported to date [4]. We performed a comprehensive literature review to identify cases of *S. maltophilia* meningitis (Table 3).

**Table 3:** . Cases with S. maltophilia meningitis F: female, M: male, TMP-SXM: Trimethoprim- sulfamethoxazole, IVH: intraventricular haemorrhage, SCT: stem cell transplant, EVD: external ventricular drain, VPS: ventriculo peritoneal shunt,  HTN: hypertension, SAH:  subarachnoid haemorrhage   *duration not known

Year of publication	Age/gender	Prior Neurosurgical interventions	Comorbid illnesses	Therapy (Duration )	Outcome
Patrick et al 1975 [5]	70 years/M	None	Emphysema	Sulphamidine and chloramphenicol (12 days)	Cured
Denis et al, 1977 [6]	13 months/F	None	None	Chloramphenicol and sulfadoxime/duration*	Cured
Denis et al, 1977 [6]	8 months/M	None	None	Ampicillin and colistin*	Died
Trump et al, 1982 [7]	52 years/F	Ommaya reservoir	Metastatic malignancy	Chloramphenicol and gentamicin*	Cured
Dewi et al, 1984 [8]	Preterm infant/M	Yes	IVH	none	Died
Muder et al, 1987 [9]	65 years/M	EVD	IVH	TMP-SXM*	Cured
Girijaratnakum-ari et al, 1993 [10]	28 years/F	craniotomy	CP angle tumor	I/V ciprofloxacin (3 weeks)	Cured
Nguyen et al,1994 [11]	64 years/M	VPS	Meningioma	I/V TMP-SX with intrathecal gentamicin*	Cured
Papadakis et al, 1997 [12]	36 years/F	Ommaya reservoir	Melanoma	TMP-SXM*	Cured
Papadakis et al, 1997 [12]	41 years/M	Ommaya reservoir	Autologous SCT	TMP-SXM*	Died
Spencer et al, 2001 [13]	31 years/F	No	None	TMP-SXM (14 days)	Cured
Caylan et al,2002 [14]	52 years/M	VPS	SAH	TMP-SXM DS (3 weeks)	Cured
Platsouka et al, 2002 [15]	42 years/M	Yes	Malignancy	TMP-SXM with ceftazidime *	Cured
Lo et al, 2002 [16]	Preterm infant/F	No	None	Ciprofloxacin (4 weeks)	Cured
Libanore et al, 2004 [17]	49 years/M	No	None	Amikacin with ceftazidime (14 days )	Cured
Reddy et al , 2006 [18]	48 years/M	craniotomy	Pituitary tumor	TMP-SXM (12 days )followed by moxifloxacin (21 days)	Cured
Yemisen et al , 2008 [2]	30 years/M	Craniotomy	Subdural hematoma	Ciprofloxacin and TMP-SXM (14 days)	Cured
Rojas et al , 2009 [19]	Preterm/M	Yes	Nil	Ciprofloxacin and TMP-SXM (21 days)	Cured
Huang et al ,2013 [4]	48 years/M	Craniotomy and EVD	Nil	IV TMP-SXM + IV ciprofloxacin (23 days)	Cured
Huang et al ,2013 [4].	61 years/M	craniotomy and EVD	Nil	IV ceftazidime (63 days ) → IV ciprofloxacin (35 days )	Cured
Huang et al , 2013 [4].	43 years/F	craniotomy and EVD	Intracranial artery aneurysm	Oral TMP-SXM (33) + IV levofloxacin (11)→ IV ciprofloxacin (22)	Cured
Huang et al,2013 [4]	48 years/F	craniotomy and EVD	intracranial artery aneurysm	IV TMP - SXM + IV moxifloxacin (42 days)	Cured
Huang et al, 2013 [4]	62 years/F	craniotomy and EVD	DM	IV SMZ–TMP (61)+ IV moxifloxacin (42) (28)	Died
Huang et al,2013 [4]	45 years/M	craniotomy and VP shunt	Pituitary tumor	IV moxifloxacin → IV moxifloxacin+ oral TMP-SXM (14)	Died
Wang et al, 2013 [3]	73 years/M	Yes, EVD	Nil	Ceftazidime (21 days)	Cured
Wang et al, 2013 [3]	61 years/M	Neuro endoscopy	Nil	Ceftazidime (21 days)	Cured
Correia et al, 2014 [20]	4 years/M	EVD	Nil	TMP-SMX + ceftazidime + levofloxacin 21 days	Cured
Ibrahim et al,2018 [1]	13 days old/M	No	Nil	TMP-SMX and Ciprofloxacin (21 days)	Cured
Present case	35 years/M	Yes /VP shunt	Nil	TMP-SMX and ceftazidime (21 days)	Cured
Present case	60 years/F	Yes/EVD	HTN	TMP-SMX and colistin – (21 days)	Cured

Among the 30 reported cases of *S. maltophilia* meningitis, there was a male predominance (67.7%) and only seven cases (22.5%) belonged to pediatric age groups. In the current review, 87% of *S. maltophilia* meningitis cases were hospital-acquired and a majority of patients had undergone neurosurgical intervention prior to the onset of meningitis. Prolonged antibiotic therapy (e.g. carbapenem, antipseudomonal cephalosporin, and aminoglycosides ), healthcare exposure, and prior neurosurgical procedures were the major risk factors (in more than 50% of the cases) associated with *S. maltophilia* infection in healthcare settings [4]. Clinicians should suspect *S. maltophilia* meningitis in patients with a previous history of hospitalization, presence of devices like VP shunt, neurosurgical procedure, and prior prolonged antibiotic exposure.

The management of infections caused by *S. maltophilia* is challenging due to its intrinsic resistance to multiple antibiotics, the difficulty of in vitro testing antimicrobial susceptibility, and the development of resistance during treatment. *S. maltophilia* has multiple mechanisms of resistance including the production of beta-lactamase, decreased membrane permeability to antibiotics, the presence of efflux pumps, and antibiotic modifying enzymes [11]. The optimal antibiotics to treat *S. maltophilia* infections are based on in vitro studies, anecdotal evidence, and only a few clinical trials.

The antibiotic of choice for *S. maltophilia* is TMP-SMX, which is based on good clinical outcomes and susceptibility profiles reported from previous studies [2,4]. Furthermore, TMP-SMX is the most frequently used antibiotic, both alone or in combination, in previously reported cases of *S. maltophilia* meningitis [1,2,4,9,11-15,18-20]. Both of our patients were treated with TMP-SMX (in combination with ceftazidime in one patient, and colistin in the other) based on susceptibility data.

Other antibiotics reportedly used in combination with TMP-SMX for *S. maltophilia* meningitis are quinolones (ciprofloxacin, levofloxacin, and moxifloxacin), ceftazidime, and polymyxin. Among the 19 cases from the current review that were treated with TMP-SMX, the majority received combination therapy.

There is no guideline available for the management of severe infection caused by *S. maltophilia*. Due to its resistance to multiple antibiotics, TMP-SMX is used in combination with other antimicrobial agents for the treatment of severe infection; although, there is lack of clinical data regarding the efficacy of combination therapy [2]. There was no difference found in the clinical outcomes of patients with *S. maltophilia* meningitis, either when treated with TMP-SMX alone or in combination therapy. The duration of therapy was between 14 and 21 days. Preferably, devices like EVD, VP shunt should be removed to achieve microbiological clearance.

The overall mortality rate of patients with *S. maltophilia* meningitis was 16% in the present review but high mortality was observed in pediatric patients in comparison to adults (28.5% vs 12.5%). *S. maltophilia* meningitis poses a treatment challenge due to the limited number of effective antibiotics available.

To the best of our knowledge, this is the first case series of *S. maltophilia* meningitis from our country. Both cases were managed successfully with antibiotics along with replacement of intraventricular devices and had good clinical outcomes.

## Conclusions

*S. maltophilia* is an extremely rare but emerging cause of nosocomial meningitis and is strongly associated with prior hospitalization, prolonged use of antibiotics, presence of devices, and neurosurgical intervention. The optimal therapy for *S. maltophilia* meningitis is not well defined and remains a therapeutic challenge. Trimethoprim-sulfamethoxazole is the drug of choice and duration of therapy is 14 to 21 days. TMP-SMX-based combination therapy depending upon antimicrobial susceptibility data is recommended for *S. maltophilia* meningitis with good clinical outcome. The overall mortality rate of patients with *S. maltophilia* meningitis was low in this review, with pediatric patients having high mortality in comparison to adults.

*S. maltophilia* must be considered as a potential pathogen in cases of nosocomial meningitis developed after neurosurgical intervention, prolonged hospitalization, antibiotic use, and presence of indwelling devices.
